# An EEG motor imagery dataset for brain computer interface in acute stroke patients

**DOI:** 10.1038/s41597-023-02787-8

**Published:** 2024-01-25

**Authors:** Haijie Liu, Penghu Wei, Haochong Wang, Xiaodong Lv, Wei Duan, Meijie Li, Yan Zhao, Qingmei Wang, Xinyuan Chen, Gaige Shi, Bo Han, Junwei Hao

**Affiliations:** 1 Department of Neurology, Xuanwu Hospital Capital Medical University, Beijing, 100053 China; 2 National Center for Neurological Disorders, Beijing, 100053 China; 3 Department of Neurosurgery, Xuanwu Hospital Capital Medical University, Beijing, 100053 China; 4 The Key Laboratory of Biomedical Information Engineering of Ministry of Education, Institute of Health and Rehabilitation Science, School of Life Science and Technology, Xi’an Jiaotong University, Xi’an, Shanxi 710049 China; 5 Department of Psychology, University of Chinese Academy of Sciences, Beijing, 100000 China; 6 Stroke Biological Recovery Laboratory, Department of Physical Medicine and Rehabilitation, Spaulding Rehabilitation Hospital, Harvard Medical School, Charlestown, MA 02129 USA; 7 Donders Institute of Cognition and Behaviour, Radboud University Medical Center, Nijmegen, The Netherlands; 8 Chinese Institute for Brain Research, Beijing, 100053 China

**Keywords:** Stroke, Scientific data, Databases

## Abstract

The brain-computer interface (BCI) is a technology that involves direct communication with parts of the brain and has evolved rapidly in recent years; it has begun to be used in clinical practice, such as for patient rehabilitation. Patient electroencephalography (EEG) datasets are critical for algorithm optimization and clinical applications of BCIs but are rare at present. We collected data from 50 acute stroke patients with wireless portable saline EEG devices during the performance of two tasks: 1) imagining right-handed movements and 2) imagining left-handed movements. The dataset consists of four types of data: 1) the motor imagery instructions, 2) raw recording data, 3) pre-processed data after removing artefacts and other manipulations, and 4) patient characteristics. This is the first open dataset to address left- and right-handed motor imagery in acute stroke patients. We believe that the dataset will be very helpful for analysing brain activation and designing decoding methods that are more applicable for acute stroke patients, which will greatly facilitate research in the field of motor imagery-BCI.

## Background & Summary

Despite improvements in preventive and treatment measures, stroke still results in high morbidity, a high disability rate and a high mortality rate. It is one of the leading causes of death and results in long-term disability worldwide^1–3^. Importantly, most patients with stroke have residual motor impairment of the upper limb, which further restricts their ability to perform daily activities and imposes heavy burdens on their family and on society^4,5^. Thus, the rehabilitation of upper limb function is an important treatment goal for stroke patients^2^.

In recent years, neural prostheses that allow devices to communicate directly with parts of the brain, brain-computer interfaces (BCIs), have been rapidly developed and are widely used in the medical field^6,7^. BCIs, a new treatment method, are used to rehabilitate upper limb function; thus, BCIs can facilitate the rehabilitation of stroke patients^6–8^. Brain data for the development of BCIs can be obtained with multiple techniques, including electroencephalography (EEG), magnetoencephalography (MEG), functional magnetic resonance imaging (fMRI) and near infrared spectroscopy (NIRS)^9^. Among these techniques, EEG is widely used because of its non-invasive nature, high temporal resolution, portability and relatively low cost^8,10–12^.

Motor imagery (MI) involves imagining the performance of motor activities, resulting in changes in activity in the corresponding motor cortex; this is an important paradigm for EEG-based BCI that has wide applications in the fields of neurorehabilitation and neuroprosthetics^10,11,13,14^. However, due to the dynamic nature of EEG signals, the features must be extracted and a classifier must be trained by decoding an EEG dataset for the BCI before use^15^. Regarding the features used for EEG decoding, the common spatial pattern (CSP) is an optimal set of spatial filters for projection and is commonly used^16–19^. Activity in multiple frequency bands is extracted from EEG signals, which represent different brain activities^20–23^. Multifeature fusion extraction under multiple frequency bands was proposed to improve the accuracy of decoding MI data^20,21,24^. For machine learning and deep learning, MI decoding algorithms generally need to learn and validate the robustness of the model on numerous datasets obtained from various subjects (healthy subjects and stroke patients).

Notably, some MI datasets are already publicly available, such as EEG Self-Paced Key Typing^25^, EEG Synchronized Imagined Movement^25^, datasets Ia^26^, BCI competition III^27^, BCI competition IV^24^, and other related datasets^28–32^. However, these datasets were collected from healthy people rather than stroke patients and hence are limited in clinical applications. Other limitations of these existing datasets include short recording times, small sample sizes and small numbers of BCI signals^6,12,24–34^. These limitations not only indicate that the accuracy of datasets need to be improved but also the need for a dataset that can be used for research, development, and application of BCIs in stroke patients, especially in patients with upper limb dysfunction. Therefore, expanding the EEG datasets for BCI to restore upper limb function in stroke patients is crucial.

In this paper, we collected data from 50 acute stroke patients to create a dataset containing a total of 2,000 ( = 50 × 40) hand-grip MI EEG trials. Subjects completed specific MI tasks according to on-screen prompts while their EEG data were recorded. The public dataset consists of four sets of data: the MI guide material, raw EEG data, pre-processed data (directly used to categorize the trial data), and patient characteristics. We have provided these data so that researchers can reuse the dataset as they see fit.

With this dataset, we initially compared EEG data acquired during left- and right-handed MI in acute stroke patients and performed a binary decoding task using existing baseline data and state-of-the-art methods to demonstrate that the collected EEG data could be classified according to hand used^35,36^. Also, we proposed the optimal time window and filterbank discriminant geodesic filtering and minimum distance to mean (TWFB + DGFMDM) to improve the decoding performance. In this dataset, our proposed method obtained a decoding accuracy of 72.21%. We expect that our dataset will greatly facilitate MI-BCI research on brain activation and can inform clinical rehabilitation programs for stroke patients.

## Methods

### Participants

This dataset includes data from 50 acute stroke patients (the time after stroke ranges from 1 day to 30 days) admitted to the stroke unit of Xuanwu Hospital of Capital Medical University. The patients included 39 males (78%) and 11 females (22%), aged between 31 and 77 years, with an average age of 56.70 years (SD = 10.57) (shown in Table 1). Twenty-three patients (46%) had right hemiplegia, 27 patients (54%) had left hemiplegia. The average National Institute of Health Stroke Scale (NIHSS) score was 4.16 (SD = 2.85), the Modified Barthel Index (MBI) score was 70.94 (SD = 18.22), and the modified Rankin Scale (mRS) score was 2.66 (SD = 1.44). All participants and their families were informed about the purpose and the procedures of the experiments. Informed consent for the collection and publication of data was obtained. All experiments were approved by the Ethics Committee of Xuanwu Hospital of Capital Medical University (No. 2021-236).

**Table 1:** Baseline characteristics of subjects.

Information of patients	Value
Sex (female/male)	11/39
Age (years; months)	56.70(SD = 10.57)
Duration	5.78(SD = 6.90)
Cerebral Infarction	50
Paralysis Limb (left/right)	27/23
NIHSS	4.16(SD = 2.85)
MBI	70.94(SD = 18.22)
mRS	2.66(SD = 1.44)

Data shown are the mean (SD) or n (%). MBI: Modified Barthel Index. mRS: modified Rankin Scale. NIHSS: National Institute of Health Stroke Scale.

### Experimental procedures

**Fig. 1:**
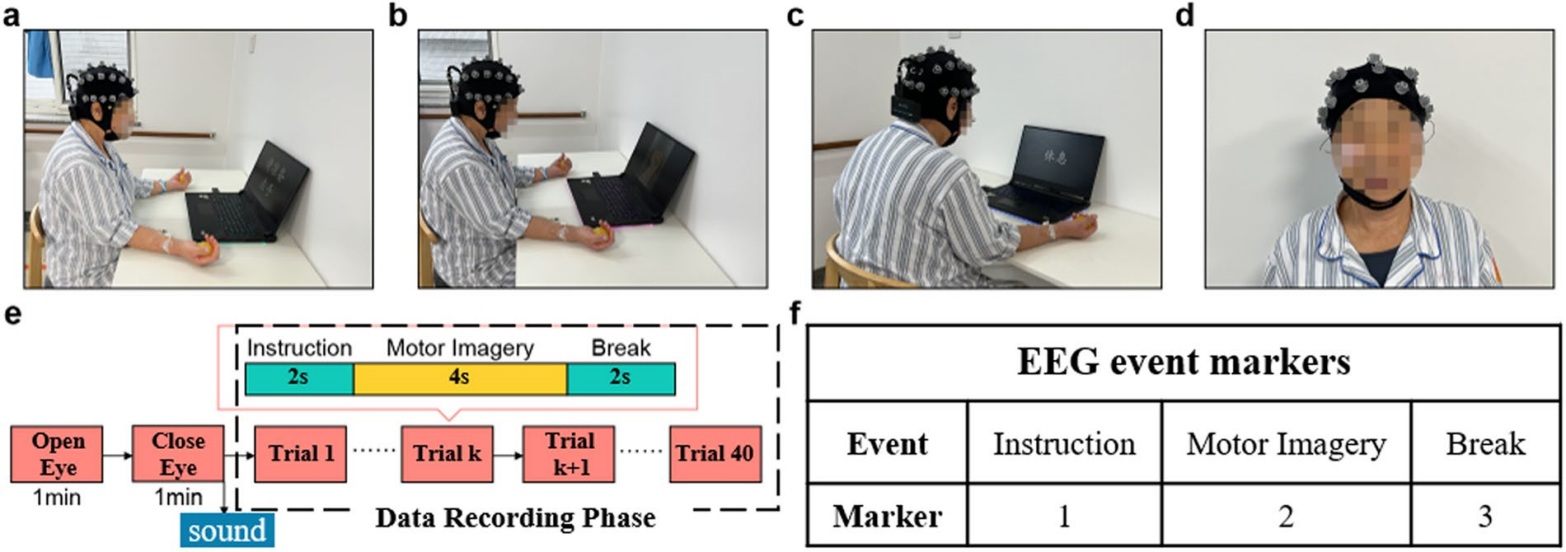
Experimental procedures. Informed consent was obtained from the individual in the figure for the publication of the images. (**a**–**c**) The process used to collect data during left- and right-hand motor imagery. (**d**) Horizontal and vertical EOG data were collected. (**e**) One trial of the MI experiment. (**f**) EEG event markers.

The experimental procedure for all participants is shown in Fig. 1. Before the start of the experiment, the subject sat in a chair in a position as comfortable as possible with an EEG cap placed on their head; subjects were positioned approximately 80 cm away from a computer screen in front of them. The computer played audio instructions to the patient about the procedure.

Each experiment lasted approximately 20 minutes, including preparation time and approximately 10 minutes of signal recording. Before the start of the MI experiment, the patients opened their eyes and closed their eyes for 1 minute each. The MI experiment was divided into 40 trials, and each trial took 8 seconds, which consisted of three stages (instruction, MI and break), as shown in Fig. 1e. In the instruction stage, patients were prompted to imagine grasping a spherical object with the left- or right-hand, as shown in Fig. 1a. In the MI stage, participants imagined performing this action, a video of gripping motion is played on the computer, which leads the patient imagine grabbing the ball. This video stays playing for 4 s. Patients only imagine one hand movement, as shown in Fig. 1b. In the break stage, participants were allowed to relax and rest, as shown in Fig. 1c.

The MI experiments alternated between the left- and right-hand, and the patients moved onto the next stage of the experiment according to the instructions.

### Data collection and preprocessing

**Fig. 2:**
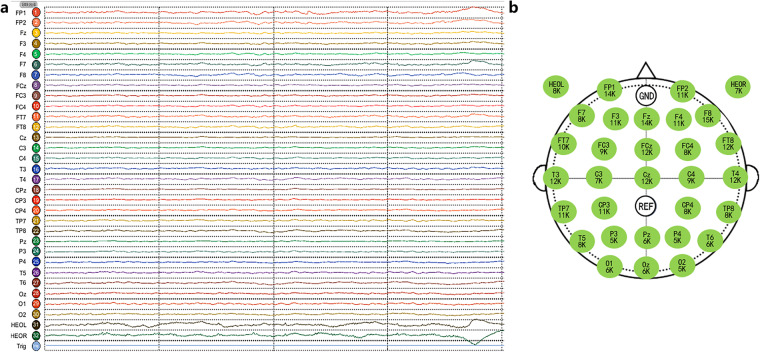
Real-time snapshot of the EEG signal and electrode impedance. (**a**) A snapshot of the raw signals recorded in 30 EEG channels and 2 EOG channels. (**b**) The impedances of all 32 electrodes were kept close to or below 20 kΩ.

The EEG data were collected through a wireless multichannel EEG acquisition system (ZhenTec NT1, Xi’an ZhenTec Intelligence Technology Co., Ltd., China), which is shown in Fig. 2. The system includes an EEG cap, an EEG acquisition amplifier, a data receiver and host computer software. The EEG cap had electrodes placed according to the international 10-10 system, including 29 EEG recording electrodes and 2 electrooculography (EOG) electrodes, as shown in Fig. 2a and Table 2. The reference electrode located at CPz position and the grounding electrode located at FPz position. All the EEG electrodes and grounding electrode are Ag/AgCl semi-dry EEG electrodes based on highly absorbable porous sponges that are dampened with 3% NaCl solution. The EOG electrodes are composed by Ag/AgCl electrodes and conductive adhesive hydrogel. The common-mode rejection ratio was 120 dB, the input impedance was 1 GΩ, the input noise was less than 0.4 µVrms, and the resolution was 24 bits. The acquisition impedance was less than or equal to 20 kΩ. The sampling frequency was 500 Hz.

**Table 2:** The relationship between channels and electrode positions.

Corresponding relationship between lead and electrode position											
Channel	1	2	3	4	5	6	7	8	9	10	11
Name	Fp1	Fp2	Fz	F3	F4	F7	F8	FCz	FC3	FC4	FT7
Channel	12	13	14	15	16	17	18	19	20	21	22
Name	FT8	Cz	C3	C4	T3	T4	CPz	CP3	CP4	FT7	TP8
Channel	23	24	25	26	27	28	29	30	31	32	
Name	Pz	P3	P4	T5	T6	Oz	O1	O2	HEOL	VEOR	

Preprocessing of the collected data was performed using the EEGLAB toolbox of MATLAB (R2019b). We performed baseline removal with mean removal method and time-domain filtering on the data from 0.5 to 40 Hz. The filtered data were split into ‘trials × channels × time-samples’ format by the marker ‘1’.

### Behavioural measures

#### National institute of health stroke scale (NIHSS)

The NIHSS is an 11-item index widely used to assess the severity of acute stroke^37^. The items include level of consciousness, gaze, visual fields, facial palsy, arm and leg motor function, ataxia, sensory function, language, dysarthria, etc. Each item is rated on a range of 0–2, 0–3 or 0–4, where 0 indicates normal and the highest score (2/3/4) indicates complete loss of function or consciousness. The scores on the 11 items are added together to obtain a total score ranging from 0 to 42; higher scores indicate more severe neurological impairment.

#### Modified barthel index (MBI)

The MBI is a 10-item scale assessing activities of daily life that is widely used for patients with stroke and brain trauma^38^. The 10 activity items include the abilities to perform personal hygiene, bathing, feeding self, using the toilet, climbing stairs, dressing, controlling bowels, controlling bladder, walking unassisted and chair/bed transfers. Each activity is rated on a scale from 1 to 5 according to the amount of assistance required to perform each task (1 = “unable to perform the task”, 5 = “fully independent”). The scores for the 10 activities are added together to obtain the total score. The total score ranges from 0 (full dependence) to 100 (full independence).

#### Modified rankin scale (mRS)

The mRS is mainly used to measure the overall functional independence of patients^39^, especially in terms of the prognosis and functional recovery of stroke patients. Items are scored on a scale from 0 to 5, and a higher score corresponds to worse functional disability.

## Data Records

The data files for the large MI EEG dataset for BCI can be accessed via the Figshare data deposition service^40^, and the source files and EEG data files in this dataset were organized according to EEG-brain imaging data structure (BIDS)^41^. EEG-BIDS provides EEG data with relevant information files that record almost all the information covered the experiment, which can help users to use these datasets quickly and directly.

**Fig. 3:**
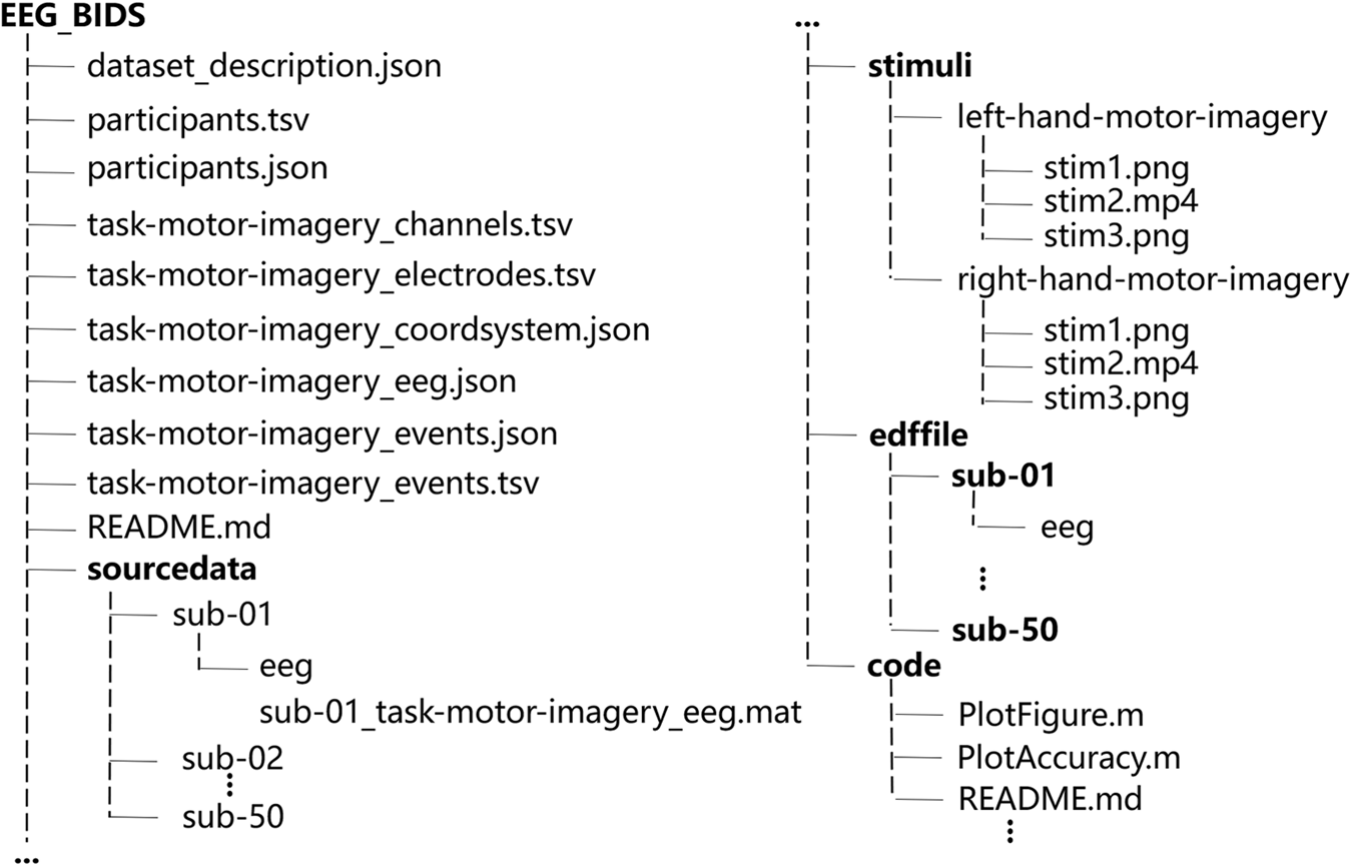
Detailed structure of datasets in the BIDS format.

The directory tree for our repository is shown in Fig. 3. The repository consists of four types of data: (1) the stimuli, which are the stimulus materials used for the experimental process; (2) the source data, which are the raw EEG data recorded from each subject during the MI task, saved as ‘.mat’ files named according to the subject number ‘*sub-xx*’; (3) the processed data, saved as ‘.edf’ files and named according to the subject number ‘*sub-xx*’; (4) and other information, including user information, electrode locations, and EEG marker events.

### Stimuli

The stimulus materials in the experiment are saved as ‘stimuli.zip’ files, and the materials used for MI of left- and right-hand actions are stored in separate folders.

### Source data

The raw EEG data from all subjects are saved as ‘sourcedata.zip’ files, and each subject has a ‘.mat’ file. The data for each subject (number: 01, 02, …, 50) are stored as a directory, and the folder naming rules are as follows: ‘sub-xx’. The file naming rules are as follows:

$$ \mbox{'} \mathrm{\text{'}sub} \mbox{-} \mathrm{xx}\_\mathrm{task} \mbox{-} \mathrm{motor} \mbox{-} \mathrm{imagery}\_\mathrm{eeg}.\,\mathrm{mat\text{'}}\mbox{'},$$

where ‘xx’ is the subject number (01, 02, …, 50), and ‘task-motor-imagery’ is the MI task. The ‘.mat’ file contains two variables:

- **rawdata:** The raw data is formatted as ‘trials × channels × time-samples’, where the number of trials is 40, the number of channels is 33, and there are 30 EEG channels (channel 18 is reference channel), 2 EOG channels (31 horizontal EOG channels and 32 vertical EOG channels), and 1 marker event channel; the number of time samples was 4,000.
- **labels:** This variable contains the task labels (“1” for left-hand MI tasks and “2” for right-hand MI tasks).

### Processed data

The raw data were imported into MATLAB (2019b) using the EEGLAB toolbox and manual preprocessing operations such as baseline removal and FIR filtering were applied. The pre-processed data of all subjects were saved in an ‘edffile.zip’ file, and those of each subject were saved in a ‘.edf’ file. It is organized according to the following rules:

$$subxx\_task \mbox{-} motor \mbox{-} imagery\_eeg.\,edf,$$

where ‘xx’ is the subject number (01, 02, …, 50) and ‘task-motor-imagery’ represents the MI task. The channel name information is saved in ‘.edf’ files.

### Code

The ‘code.zip’ file contains code for all data processing.

## Technical Validation

### Qualitative analysis

The raw EEG data of all channels from each subject were bandpass filtered in the frequency band of 8–30 Hz with a 2nd order zero-phase Butterworth filter.

#### Event-related desynchronization/synchronization (ERD/ERS)

ERD/ERS^42^ in the alpha and beta bands during MI has been extensively documented^16–19,28,30,42,43^ To infer how distinct brain areas collaborate during the execution of specific tasks, we checked ERD/ERS^44^ of both alpha and beta bands (8–30 Hz) for all subjects and plotted the curve of energy over time. The curve shows decreased power in the contralateral channel or increased power in the ipsilateral channel after the onset (on the x-axis) of MI.

The calculation method was as follows: 1) EEG data from the C3 and C4 channels from each subject were bandpass filtered in the frequency band of 8–30 Hz. 2) The square of each trial data was calculated, and the C3 and C4 channel data with the same task label were stacked and averaged according to the number of trials. 3) The average curve obtained was smoothed (smoothness coefficient = 200). 4) ERD/ERS was calculated with the following formula: ERD/ERS% = (X-M)/M × 100, where X is each time sample and M is the mean value of preparation period (−1 to 0 second, with the label ‘2’ indicating 0 seconds).

#### Time-frequency Analysis

Time-frequency analysis is a universal technique used to study the electrical activity of the brain over time and across different frequency bands. The time-frequency of central regions (electrodes C3 and C4) is highlighted here. The alpha band (8–15 Hz) and beta band (15–30 Hz) showed reduced power during MI. Continuous wavelet transforms are functions that can simultaneously capture time and frequency information, which is used to perform time-frequency analysis^44^. The detailed calculation steps are as follows: (1) selection of the wavelet basis function (Morlet wavelets), (2) signal decomposition, (3) calculation of wavelet coefficients, (4) calculation of energy or amplitude, and (5) creation of the time-frequency map.

#### Brain electrical activity mapping (BEAM)

To study the activation of the motor area, we calculated the average power distribution in the alpha and beta bands (8–30 Hz) and plotted them on a topographic map based on the channel positions. The maps generated from data during left- and right-hand MI tasks reveal a clear pattern of ERD in the contralateral motor areas.

**Fig. 4:**
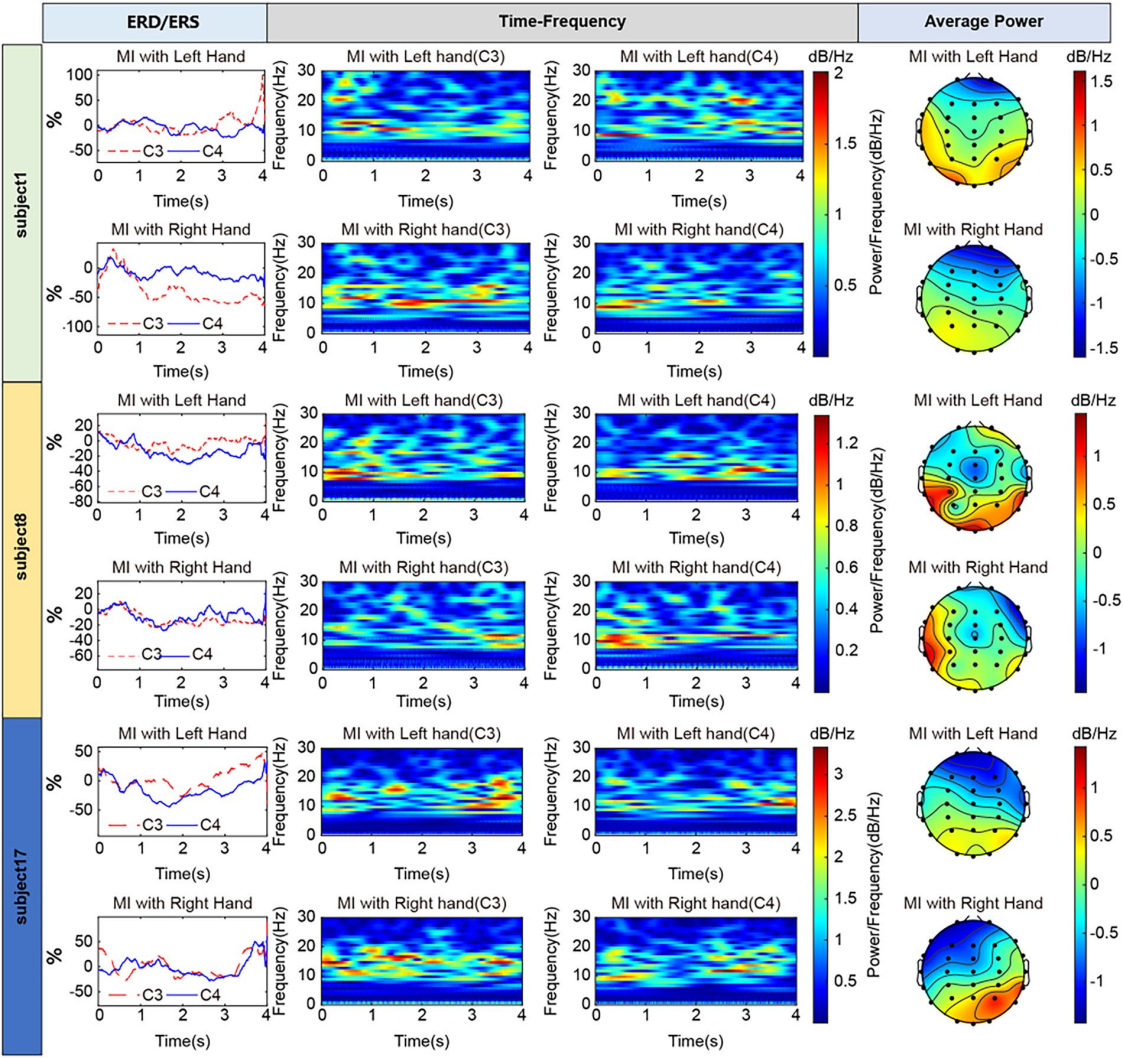
ERP, time-frequency, and average power results (on topographical maps) of three subjects.

As shown in Fig. 4, according to the power at 8–30 Hz in channels C3 and C4, the power in C3 was consistently higher than that in C4 during left-handed MI in most subjects (Subjects 1–5, 8–11, 13, 15, 18, 20, 25, 28, 29, 32, 36, 38, 40, 44, 47, and 49) (23/50 = 46%), while the power in C4 was consistently higher than that in C3 during right-handed MI. Specific subjects (2, 4, 8, 10, 11, 14, 15, 18, 19, 22, 24, 27, 28, 31, 40, 41–43, and 47–49) (21/50 = 42%) showed more significant contralateral dominance. According to the clinical characteristics of 50 patients, contralateral EEG activity during MI was more pronounced with mild to moderate hemiparesis. However, some subjects showed higher power at 8–30 Hz in C3 than in C4 when performing right-handed MI. Further analysis is needed to determine whether this finding is related to the hemiplegic side.

The EEG topography results were basically the same as those of power changes. Since EEG data were acquired from multiple channels, there might have been some deviation in the arrangement of the acquisition electrodes, which might explain why the channels with the ERD/ERS not always being C3 and C4. The results of the 50 subjects are provided in Supplementary File 1.

### Quantitative analysis

We validated the discriminability of left- versus right-hand MI EEG data to determine classification accuracy. All trials from each subject were pre-processed by high-pass filtering and referencing and then filtered both spectrally (data at 8–30 Hz) and temporally (0–4 seconds after stimulus onset).

The algorithms tested included a popular method (CSP + LDA) and a novel method based on FBCSP + SVM^20^. A second set of algorithms performed classification based on concepts from Riemannian geometry. The basic idea of these methods is that spatial covariance matrices (SCMs) of EEG epochs belonging to the Riemannian manifold of symmetric positive-definite (SPD) matrices are sampled and that the tangent space at any SPD matrix on the manifold is a finite-dimensional Euclidean space. The tested Riemannian classification methods included the minimum distance to Riemannian mean (MDRM), TSLDA^35^, Fisher discriminant geodesic filtering followed by MDRM classification (DGFMDRM^36^) and decision fusion of previous methods, including the TSLDA method and DGFMDRM method (TSLDA + DGFMDRM).

We developed a novel algorithm with the aim of combining the optimal time window and filter bank with the DGFMDRM method (TWFB + DGFMDRM). The algorithm framework is shown in Table 3.

**Table 3:** The steps performed by our algorithm for TWFB + DGFMDM.

Our Algorithm (TWFB + DGFMDM):	
Step 1	Divide the time window and filter bank, where the time window is 0–1 s, 0.5–1.5 s, 1–2 s, 1.5–2.5 s, 2–3 s, 2.5–3.5 s, 3–4 s. Divide the filter band is 8–12 Hz, 9–13 Hz, 10–14 Hz, 11–15 Hz, 12–16 Hz, 13–17 Hz, 14–18 Hz, 15–19 Hz, 16–20 Hz, 17–21 Hz, 18–22 Hz, 19–23 Hz, 20–24 Hz, 21–25 Hz, 22–26 Hz, 23–27 Hz, 24–28 Hz, 25–25 Hz, 26–30 Hz.
Step 2	Select the time window and filter bank by backtracking search optimization algorithm.
Step 3	The covariance matrix is calculated in the optimal time window and frequency window to form the covariance matrix set M.
Step 4	The local tangent space arrangement algorithm is used to reduce the feature dimension of the covariance matrix in set M.
Step 5	Input the reduced dimension characteristic matrix into discriminant geometry filter, then recognition are carried out by Riemann minimum distance.

**Fig. 5:**
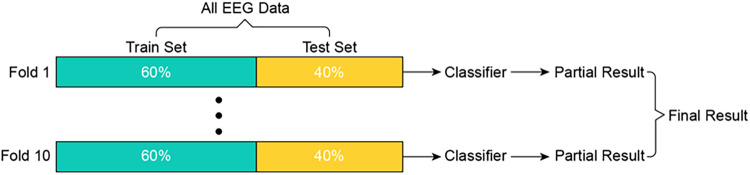
Process used to validate the findings.

The MI EEG data of each subject were divided into two sets: training data (60%) and test data (40%). To better detect differences in results, a 10-fold cross validation method was adopted^45^, which is shown in Fig. 5. The Table 4  mainly included the overall recognition accuracy of each subject and average accuracy of each of the four decoding methods, the *kappa* coefficient, which is a measure of classification consistency^46^, and the  precision and sensitivity are measures of the robustness of decoding methods. To analyse the influence of the proposed method on the detection of each type of MI from EEG data, we generated a confusion matrix consisting of the recognition results of each class for all subjects.

**Fig. 6:**
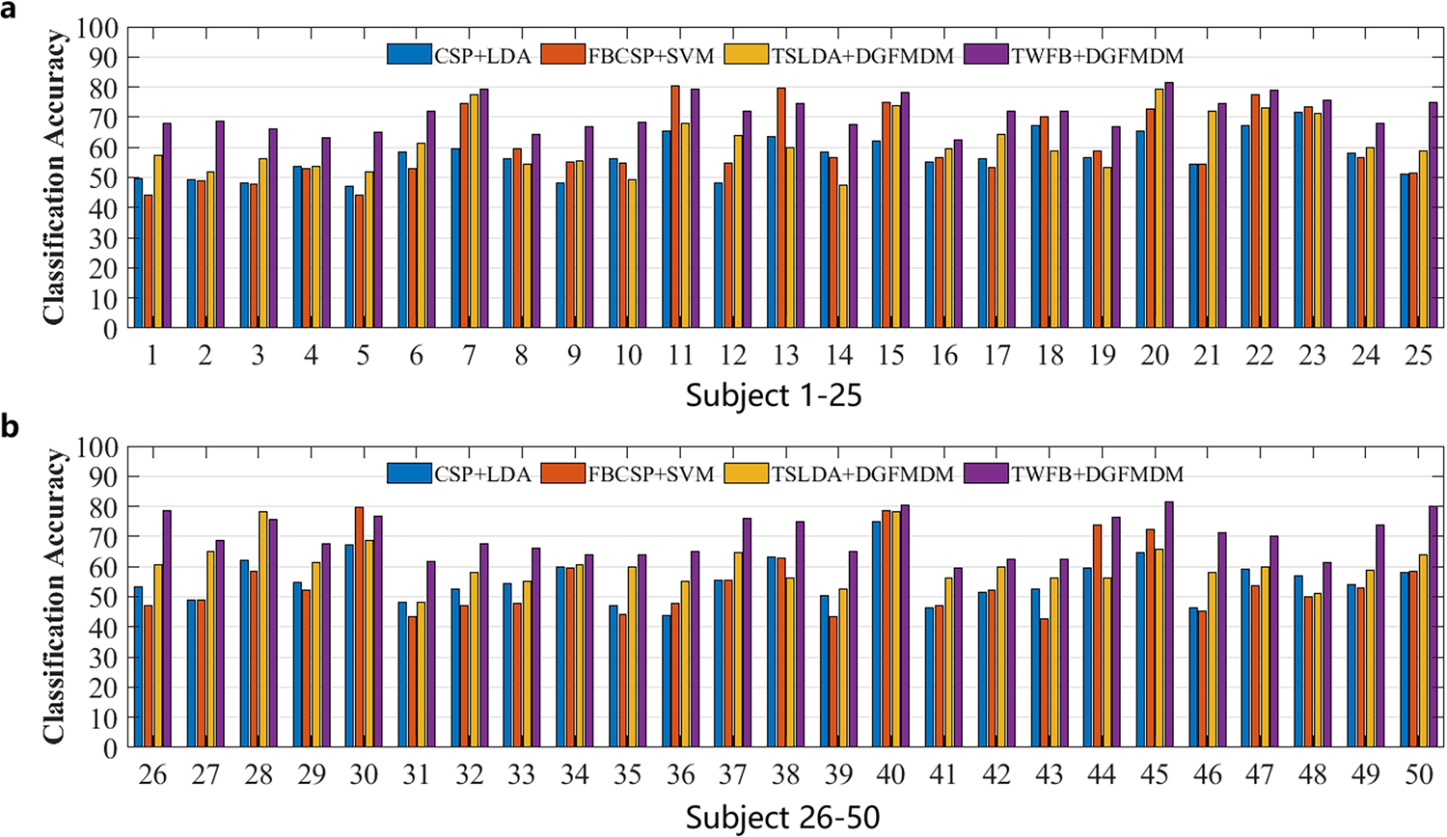
Classification accuracy in 50 subjects when using four decoding methods. (**a**) Subject 1–25. (**b**) Subject 26–50.

**Fig. 7:**
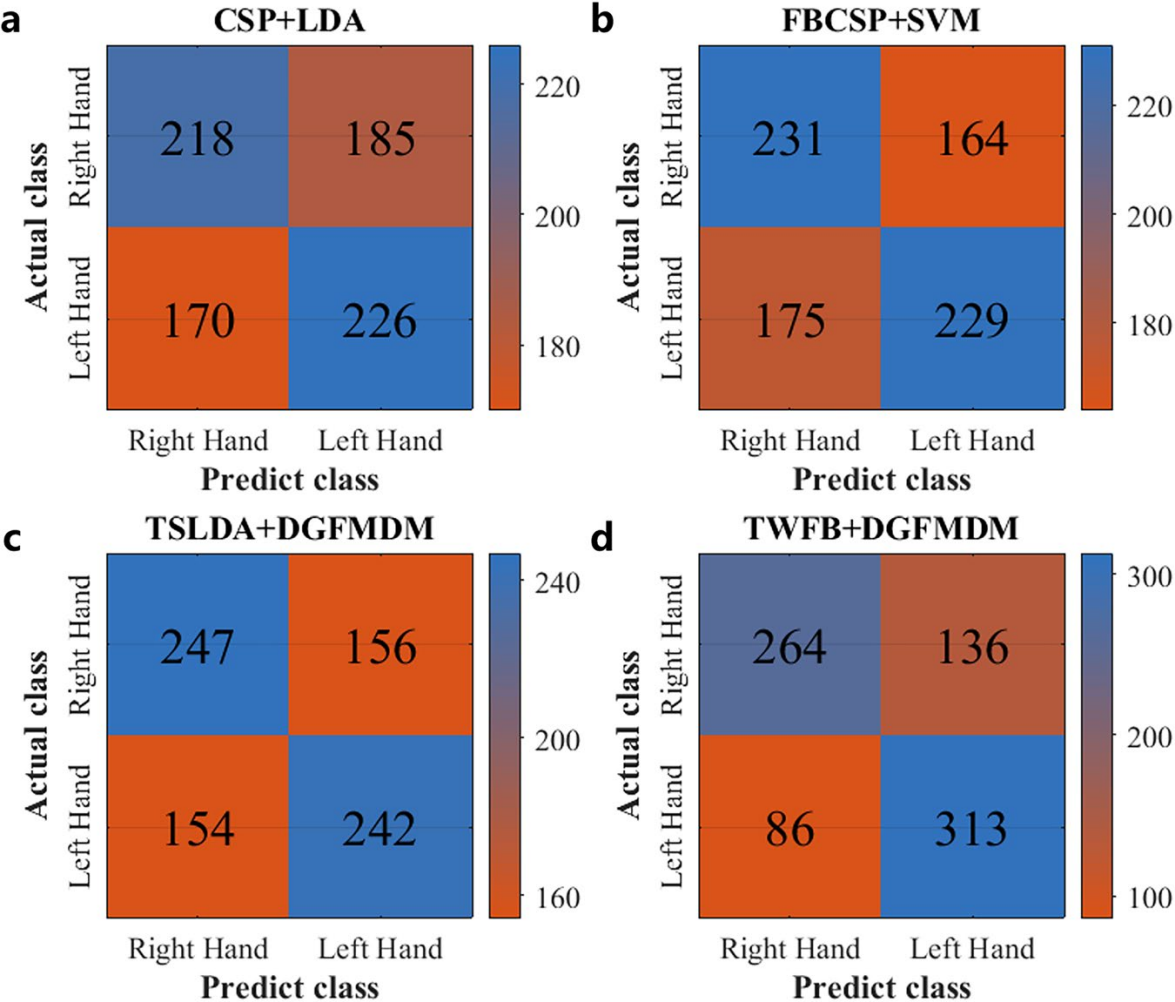
Confusion matrices of motor imagery EEG classes for the four decoding methods. (**a**) CSP + LDA, (**b**) FBCSP + SVM, (**c**) TSLDA + DGFMDM, and (**d**) TWFB + DGFMDM.

Figure 6 shows the corresponding classification accuracy results of all subjects under the four classification models. The average decoding accuracy of CSP + LDA, FBCSP + SVM, TSLDA + DGFMDRM and TWFB + DGFMDM were respectively 55.57%, 57.57%, 61.20% and 72.21%. The classification performance using this dataset also showed some differences from other common algorithms, which indicates that this dataset is reliable. As shown in Fig. 7, the horizontal axis of the confusion matrix represents the MI category (left- or right-hand movements) predicted by the recognition method, the vertical axis represents the actual MI category, the diagonal element represents the total numbers of correct classification of each MI category, and the nondiagonal element represent the total numbers of incorrect classification of each MI category.

**Table 4:** The average classification accuracy of the four models.

Method	Average Accuracy	Kappa	Precision	Sensitivity
CSP + LDA	55.57%	0.1114	0.5619	0.5707
FBCSP + SVM	57.57%	0.1514	0.569	0.5668
TSLDA + DGFMDRM	61.20%	0.224	0.616	0.6111
TWFB + DGFMDRM	72.21%	0.4442	0.7543	0.7845

## Usage Notes

Users need to be noted the following points when working with this dataset. First, it is challenging for acute stroke patients to maintain a sitting position and perform the MI paradigm, especially for some older patients. Thus, we used a task including only 20 trials per patient, and some motion artefacts were included in a part of the data (such as subjects 4, 5, 13, 14, 18, 24, 28, 33, 42, 43, 47, 48 and 49). Second, eyes-open and eyes-closed EEG data were not recorded. Final, this EEG dataset might have non-stationarity and feature covariance shift, which could refer to the study of Aleksandar, M. *et al*.^47^ and Haider, R. *et al*.^48^ to improve the classification performance.

### Supplementary information

Supplementary Information

## Data Availability

All the MATLAB scripts used in the Technical Validation section for analysis and figure generation are available in the ‘code.zip’^40^. The EEGLAB toolbox was used for analysis in MATLAB scripts. The channel_location.locs and channel_location.ced files contain the location information of channels for our EEG data and can be easily imported into EEGLAB. These scripts and files are stored in the ‘code.zip’ folder and shared with the dataset.
