# Supplementary material for: An EEG motor imagery dataset for brain computer interface in acute stroke patients
Source: Sci Data. 2024 Jan 25;11:131. doi: 10.1038/s41597-023-02787-8 (PMC10811218; doi:10.1038/s41597-023-02787-8)
Supplement: Supplementary file 1 — Supplementary Information [file 41597_2023_2787_MOESM1_ESM.pdf]

Contents

Supplementary Table 1. The detailed clinical characteristics of 50 stroke patients. 1

Supplementary Figure 1. Result about ERD/ERS, time-frequency, and average power on the topmap of subjects 1-50 (a-m). 3

a. Result about ERD/ERS, time-frequency, and average power on the topmap of subjects 1-4 3

b. Result about ERD/ERS, time-frequency, and average power on the topmap of subjects 5-8 4

c. Result about ERD/ERS, time-frequency, and average power on the topmap of subjects 9-12 5

d. Result about ERD/ERS, time-frequency, and average power on the topmap of subjects 13-16 6

e. Result about ERD/ERS, time-frequency, and average power on the topmap of subjects 17-20 7

f. Result about ERD/ERS, time-frequency, and average power on the topmap of subjects 21-24 8

g. Result about ERD/ERS, time-frequency, and average power on the topmap of subjects 25-28 9

h. Result about ERD/ERS, time-frequency, and average power on the topmap of subjects 29-32 10

I. Result about ERD/ERS, time-frequency, and average power on the topmap of subjects 33-36 11

j. Result about ERD/ERS, time-frequency, and average power on the topmap of subjects 37-40 12

k. Result about ERD/ERS, time-frequency, and average power on the topmap of subjects 41-44 13

l. Result about ERD/ERS, time-frequency, and average power on the topmap of subjects 45-48 14

m. Result about ERD/ERS, time-frequency, and average power on the topmap of subjects 49-50 15

Supplementary Table 1. The detailed clinical characteristics of 50 stroke patients										
Participant_ID	Sex(Female/Male)	Age(years)	Handedness	Time since stroke (Days)	Stroke location	Affect hand	First onset	NIHSS∗	MBI∗∗	mRS∗∗∗
sub-01	Male	45	Right	1	Left pons	Right	Yes	11	50	4
sub-02	Male	60	Right	2	Right pons	Left	Yes	3	55	4
sub-03	Male	60	Right	2	Left cerebellum, bilateral paraventricular, Right corona radiata	Left	No	3	90	1
sub-04	Male	56	Right	14	Left frontal parietal cortex, Left centrum semiovale	Right	Yes	6	90	3
sub-05	Female	44	Right	4	Left pons	Right	Yes	4	60	4
sub-06	Male	66	Left	6	Right pons	Left	No	3	85	3
sub-07	Male	62	Right	5	Left pons	Right	No	2	100	1
sub-08	Male	64	Right	5	Right basal ganglia	Left	Yes	3	85	2
sub-09	Male	57	Right	3	Left paraventricular	Right	Yes	6	55	1
sub-10	Male	55	Right	2	Right pons	Left	No	3	55	0
sub-11	Male	31	Right	7	Right paraventricular	Left	Yes	5	55	4
sub-12	Male	58	Right	1	Left medulla oblongata	Right	Yes	1	100	1
sub-13	Male	46	Right	3	Left paraventricular	Right	No	9	55	4
sub-14	Female	67	Right	2	Left pons	Right	Yes	2	75	1
sub-15	Male	63	Left	1	Right fronto-parietal temporo-occipital lobe, Right inner watershed	Left	Yes	7	55	1
sub-16	Male	57	Right	1	Left basal ganglia	Right	No	4	90	2
sub-17	Male	60	Right	3	Right paraventricular, Right basal ganglia	Left	Yes	3	85	1
sub-18	Female	60	Right	1	Right basal ganglia	Left	Yes	10	45	4
sub-19	Female	62	Right	1	Right paraventricular, Right basal ganglia	Left	No	8	40	4
sub-20	Male	34	Right	24	Left paraventricular, Right temporal lobe	Right	No	2	95	1
sub-21	Male	41	Right	5	Pons	Left	Yes	2	90	1
sub-22	Male	52	Right	6	Right temporo-parietal occipital lobe and insula, Right basal ganglia, Right paraventricular	Left	Yes	11	45	4
sub-23	Male	57	Right	2	Right paraventricular, Right basal ganglia	Left	Yes	4	40	4
sub-24	Female	55	Right	3	Left paraventricular	Right	Yes	1	60	4
sub-25	Male	47	Right	10	Right paraventricular	Left	Yes	5	55	4
sub-26	Male	61	Right	1	Right thalamus	Left	Yes	4	70	4
sub-27	Female	52	Right	5	Left basal ganglia, Left paraventricular	Right	Yes	3	70	3
sub-28	Female	42	Right	2	Left thalamus	Right	Yes	1	85	1

Participant_ID	Sex(Female/Male)	Age(years)	Handedness	Time since stroke (Days)	Stroke location	Affect hand	First onset	NIHSS∗	MBI∗∗	mRS∗∗∗
sub-29	Male	53	Right	16	Left thalamus	Right	Yes	1	95	0
sub-30	Male	68	Right	4	Right paraventricular	Left	Yes	4	100	1
sub-31	Female	59	Right	5	Left corona radiata, Left centrum	Right	No	3	80	3
sub-32	Male	74	Right	2	Left pons	Right	Yes	3	81	4
sub-33	Male	63	Right	7	Pons	Right	Yes	3	58	4
sub-34	Female	69	Right	1	Right frontal lobe	Left	No	1	85	2
sub-35	Male	69	Right	11	Right cerebellum, bilateral occipital lobes	Left	No	1	52	4
sub-36	Male	69	Right	30	Right paraventricular, Right basal ganglia	Left	Yes	6	63	3
sub-37	Male	49	Right	7	Right internal capsule	Left	Yes	3	88	4
sub-38	Male	53	Right	2	Right pons	Right	Yes	3	64	3
sub-39	Male	56	Right	2	Left cerebellar hemisphere, Left medulla oblongata	Right	Yes	11	32	5
sub-40	Female	56	Right	6	Right pons	Left	No	6	65	4
sub-41	Male	77	Right	2	Left pons	Right	No	7	60	4
sub-42	Male	54	Right	2	Left pons	Right	No	7	60	4
sub-43	Male	32	Right	3	Right frontal lobe	Left	Yes	2	65	4
sub-44	Male	59	Right	1	Right subfrontal cortex, Right basal ganglia, Right lateral ventricle, Right	Left	Yes	1	80	1
sub-45	Male	64	Right	30	Left pons	Right	Yes	6	84	4
sub-46	Male	66	Right	10	Left parietal lobe	Right	Yes	1	85	1
sub-47	Male	40	Right	4	Right medulla oblongata	Left	Yes	7	55	4
sub-48	Male	75	Right	18	Right subcortical cerebral hemisphere, Right basal ganglia, Left subparietal	Left	Yes	2	90	1
sub-49	Male	52	Right	3	Right basal ganglia	Left	Yes	1	85	1
sub-50	Female	64	Right	1	Right pons	Left	Yes	3	85	2
∗MBI: Modified Barthel Index.∗∗ mRS: modified Rankin Scale. ∗∗∗NIHSS: National Institute of Health Stroke Scale.										

Supplementary Figure 1. Result about ERD/ERS, time-frequency, and average power on the topmap of subjects 1-50 (a-m).

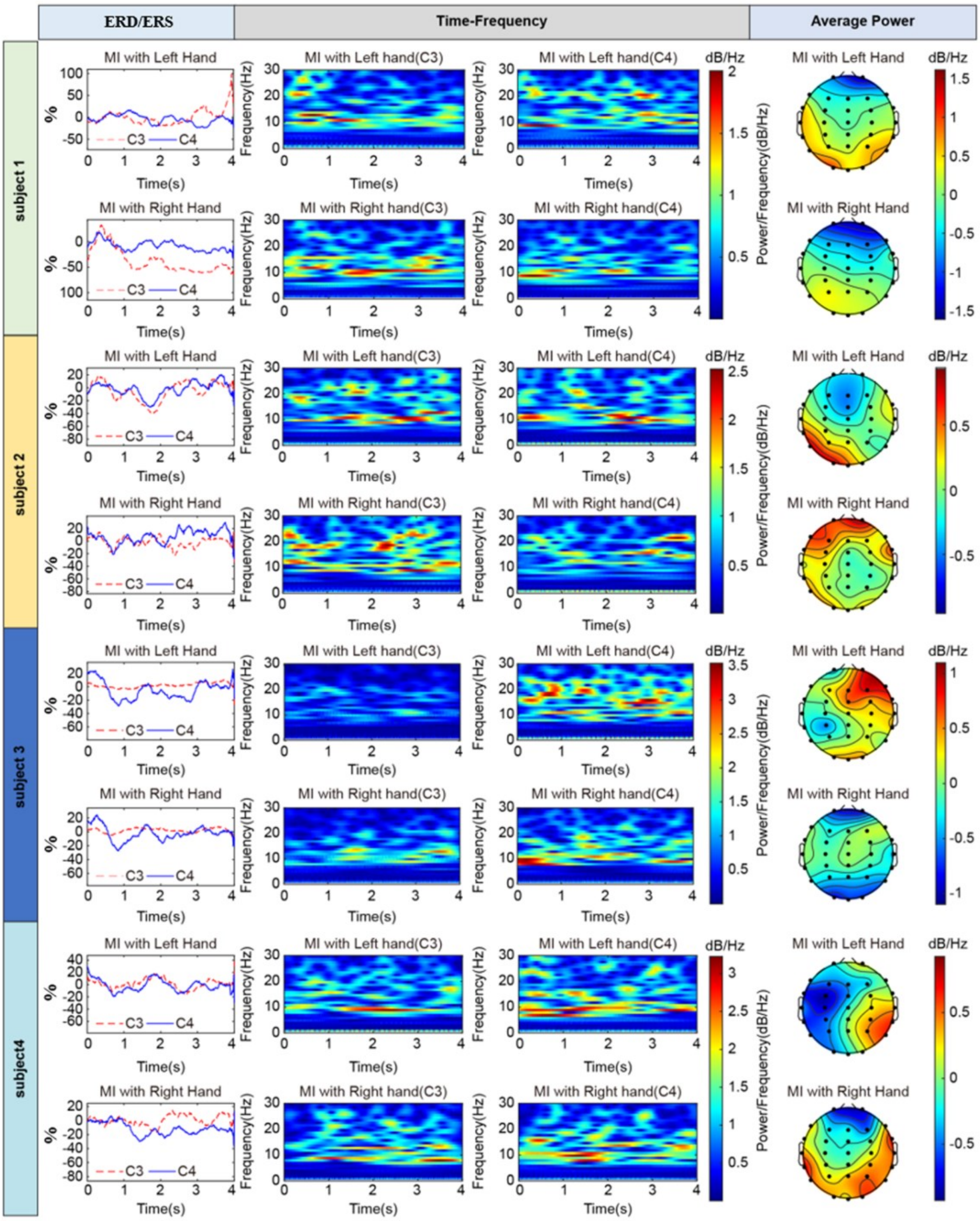

a. Result about ERD/ERS, time-frequency, and average power on the topmap of subjects 1-4

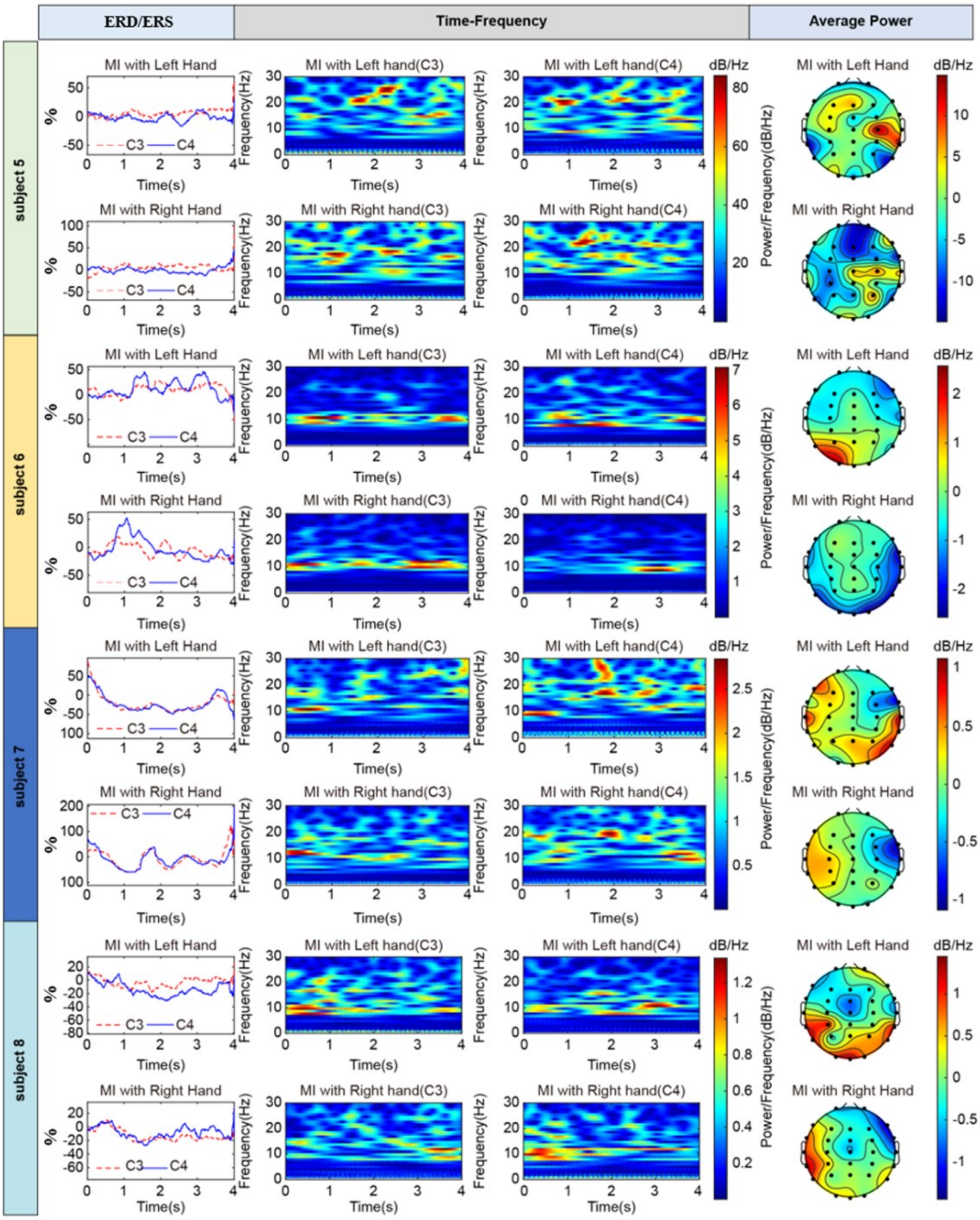

b. Result about ERD/ERS, time-frequency, and average power on the topmap of subjects 5-8

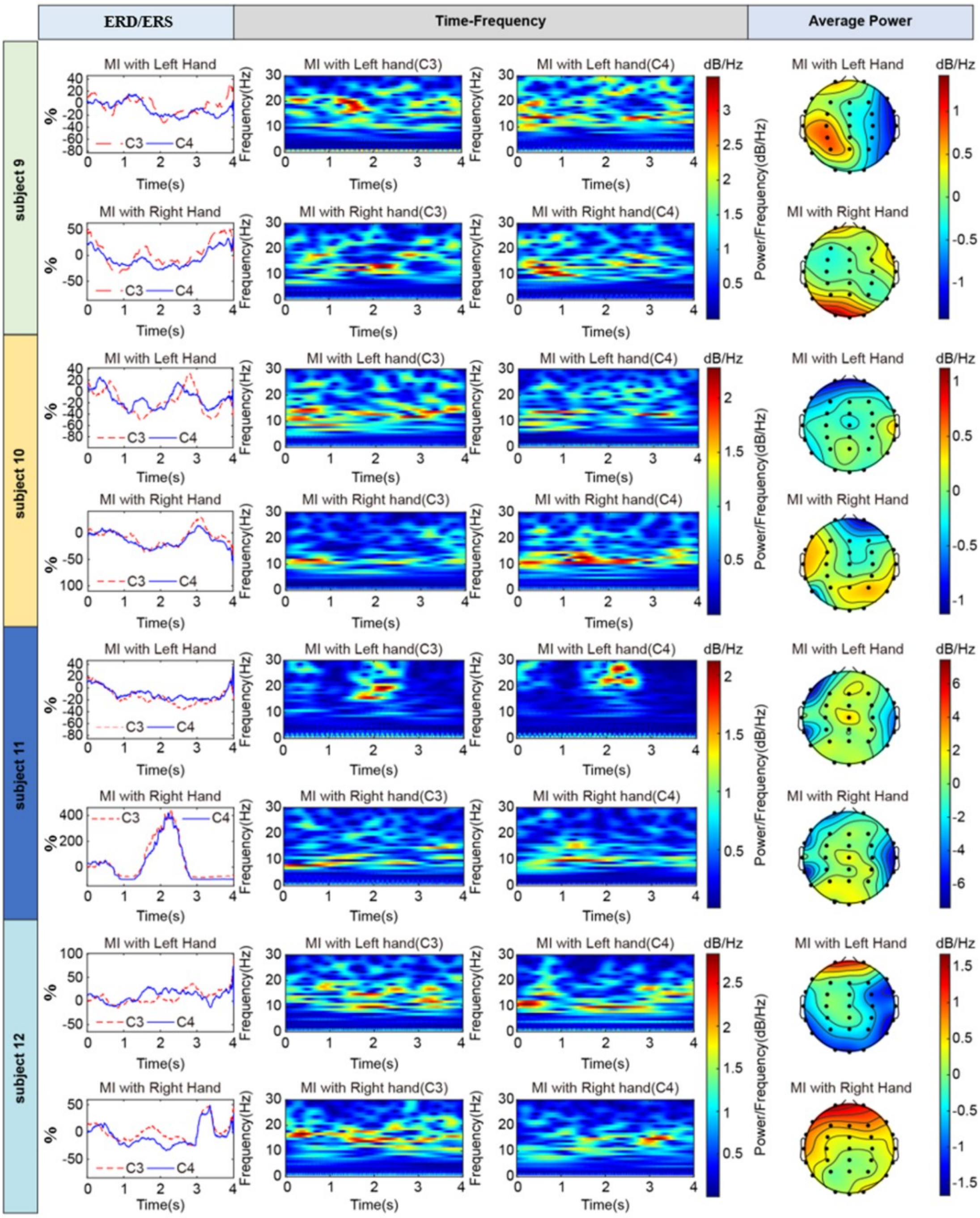

c. Result about ERD/ERS, time-frequency, and average power on the topmap of subjects 9-12

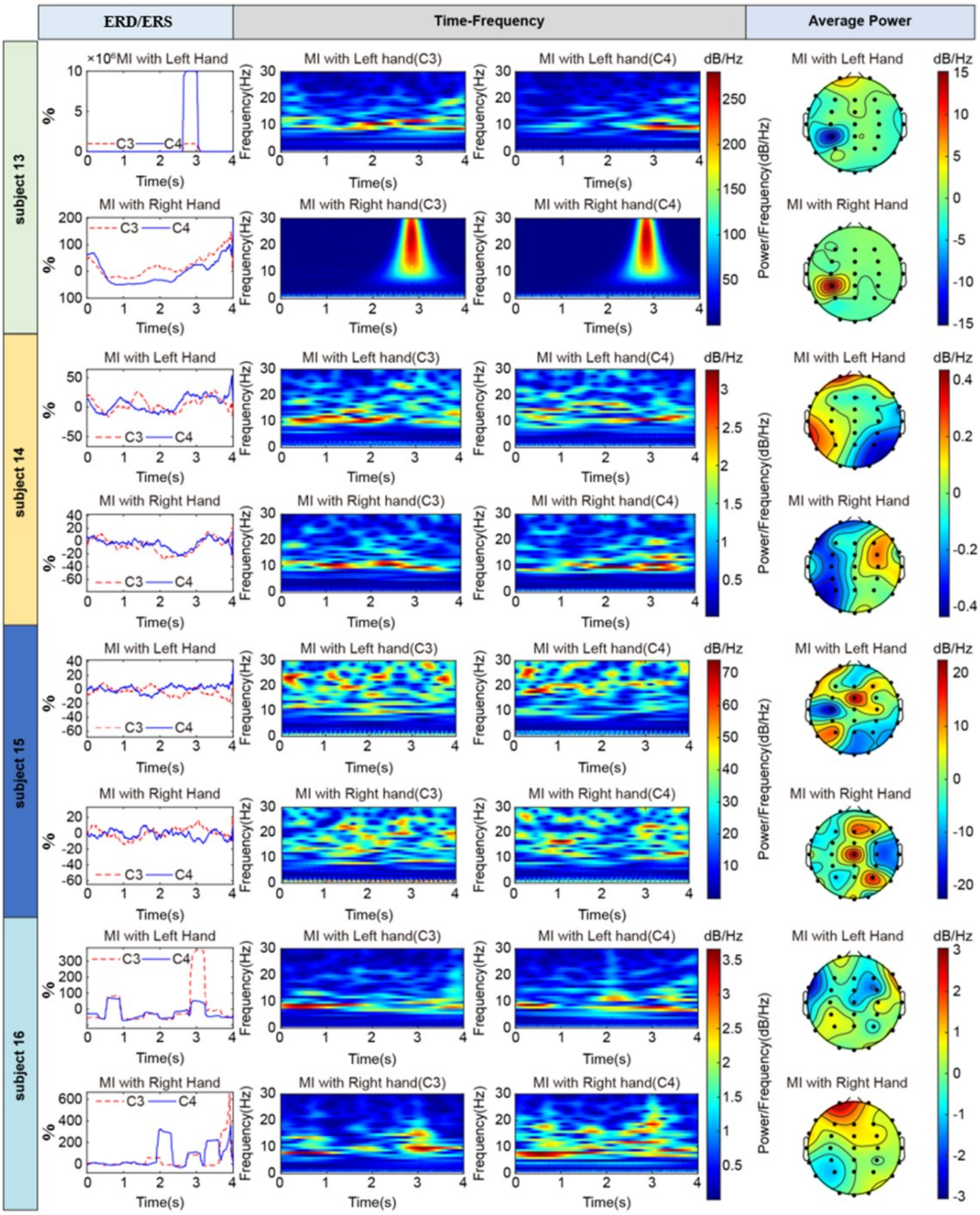

d. Result about ERD/ERS, time-frequency, and average power on the topmap of subjects 13-16

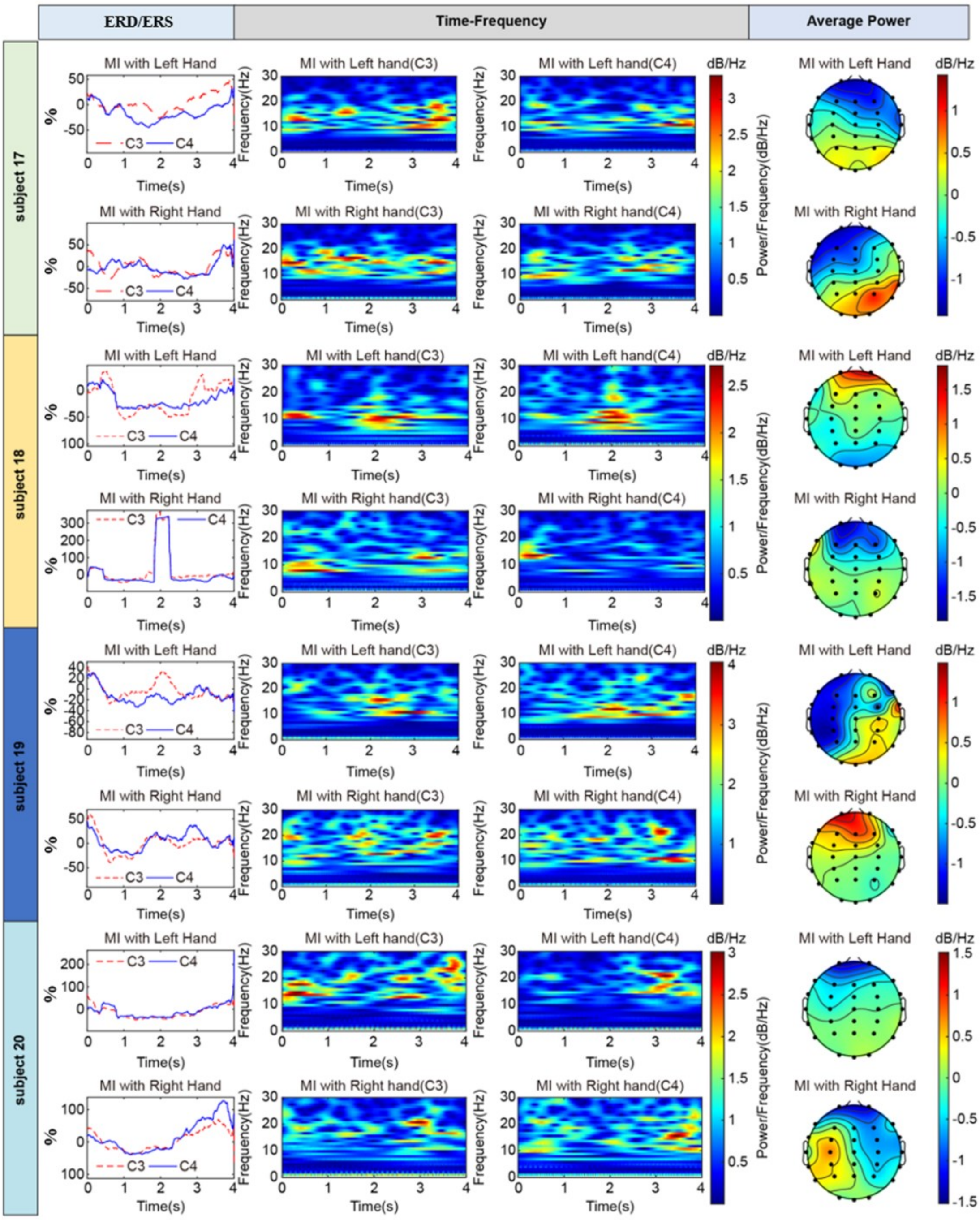

e. Result about ERD/ERS, time-frequency, and average power on the topmap of subjects 17-20

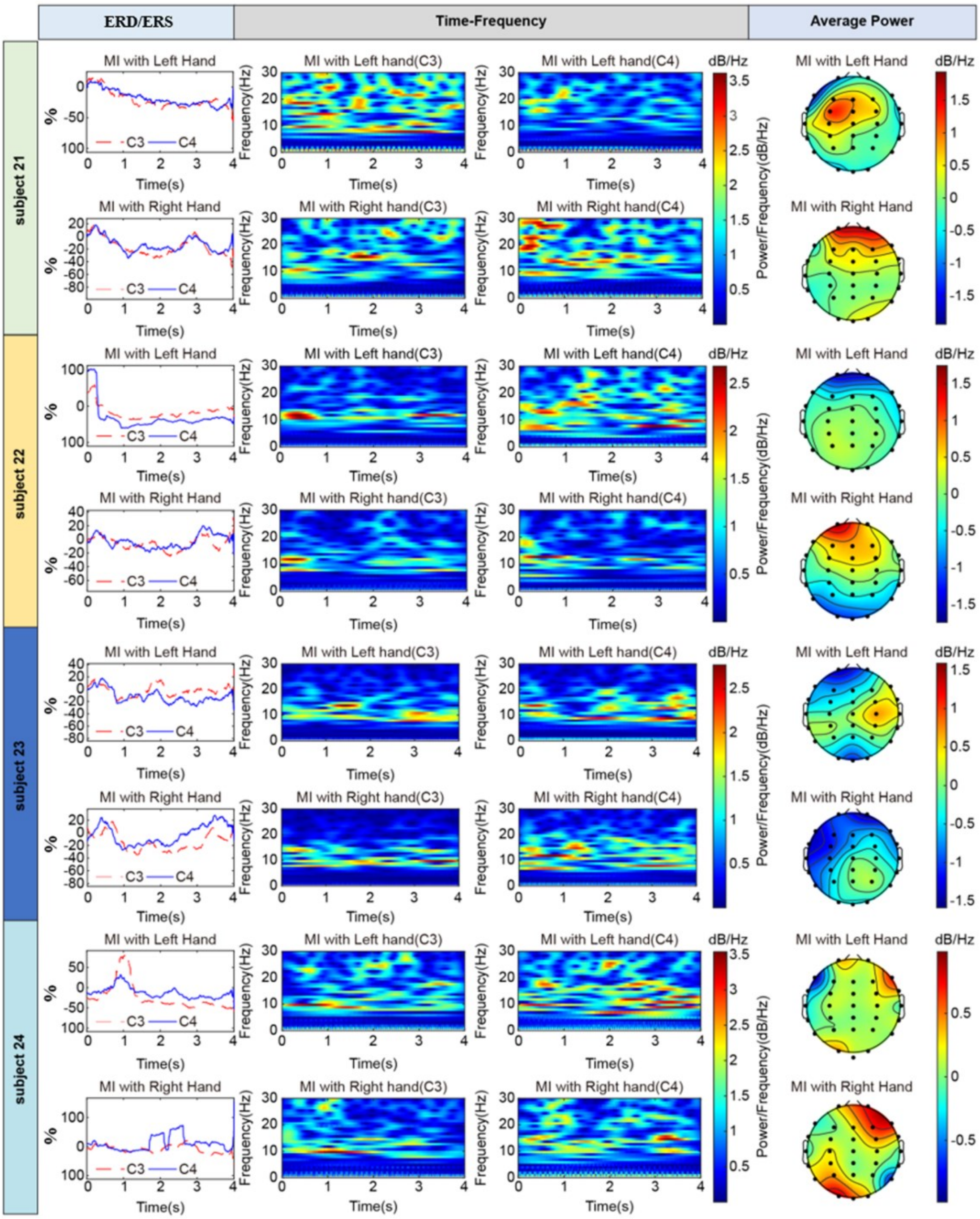

f. Result about ERD/ERS, time-frequency, and average power on the topmap of subjects 21-24

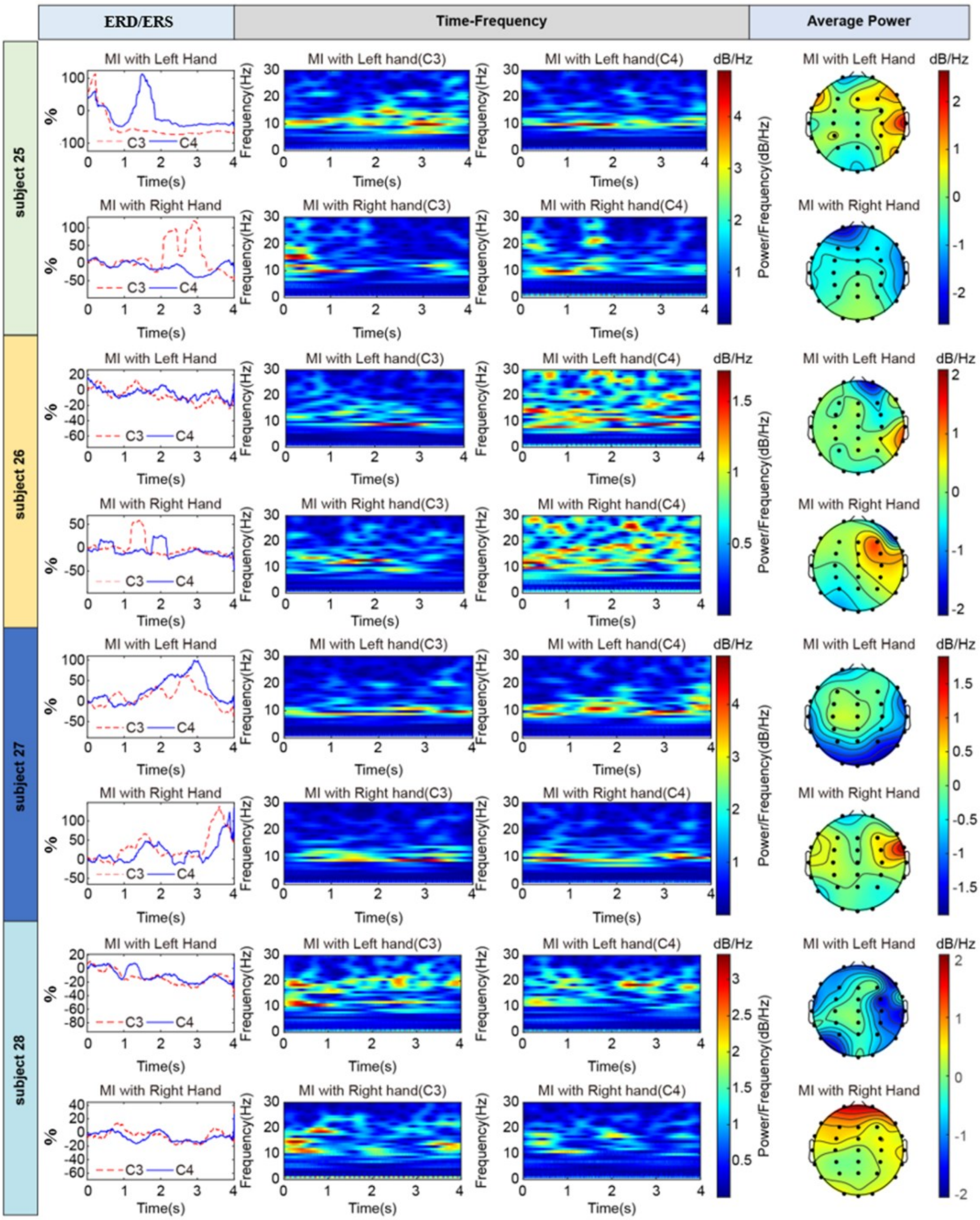

g. Result about ERD/ERS, time-frequency, and average power on the topmap of subjects 25-28

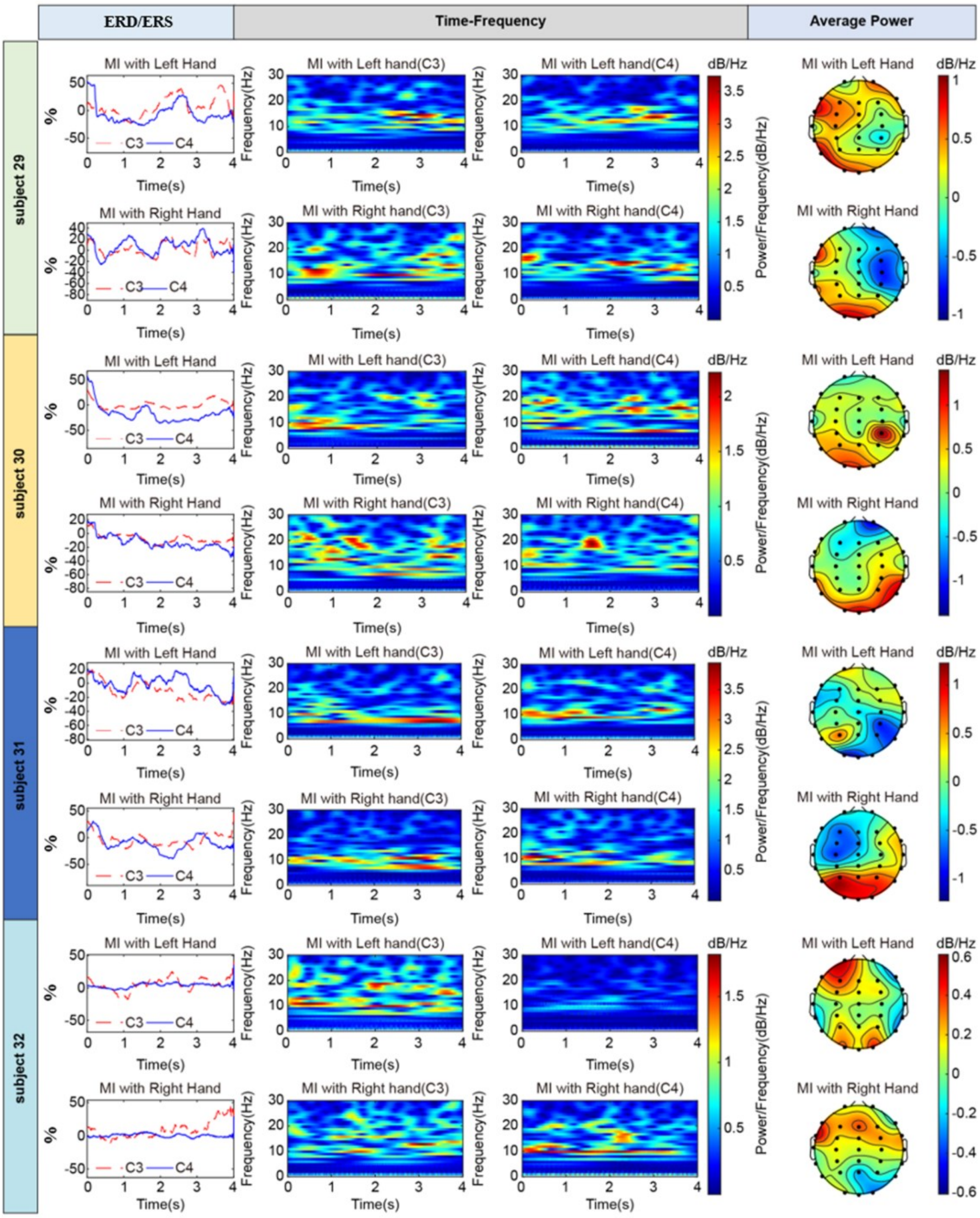

h. Result about ERD/ERS, time-frequency, and average power on the topmap of subjects 29-32

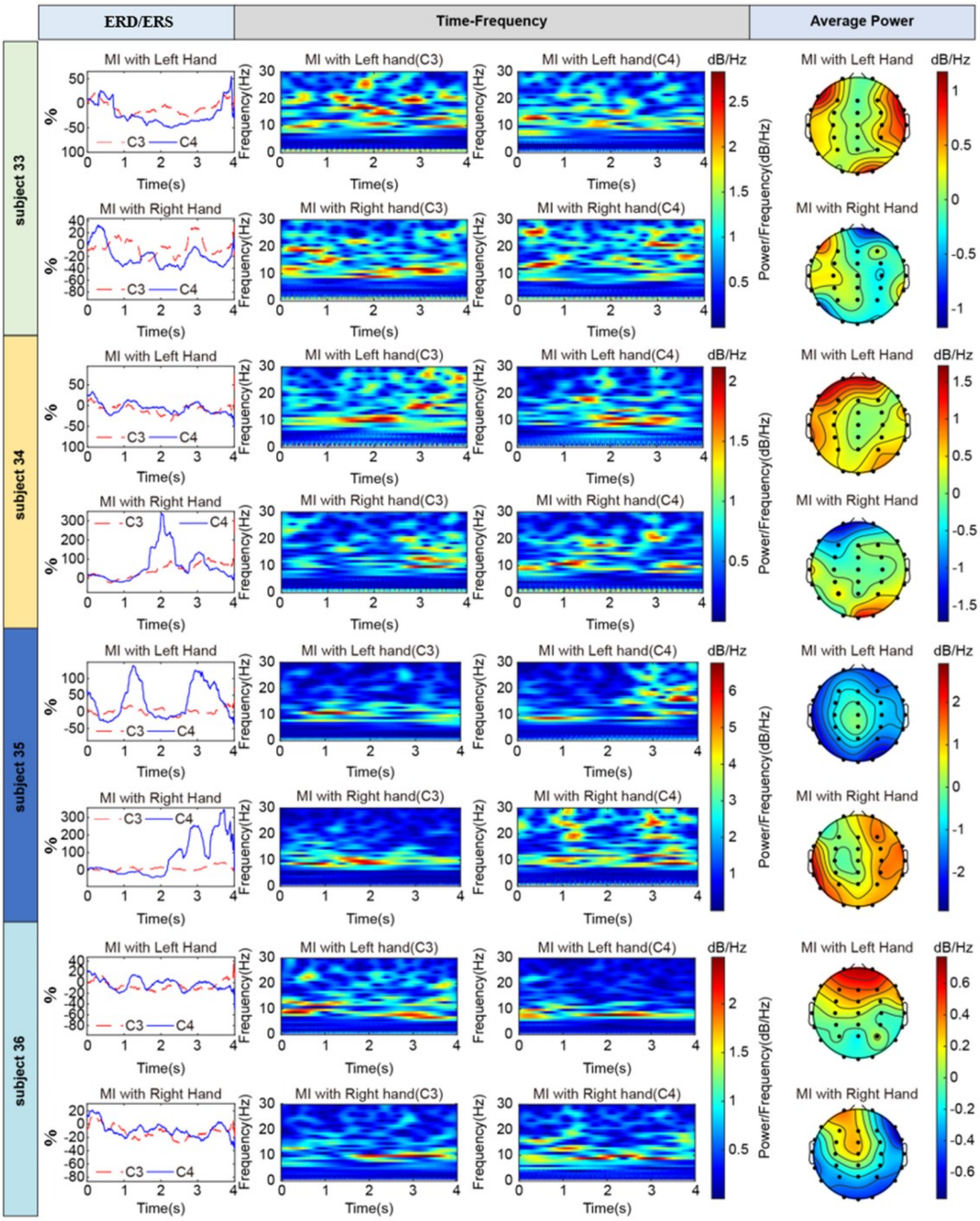

I. Result about ERD/ERS, time-frequency, and average power on the topmap of subjects 33-36

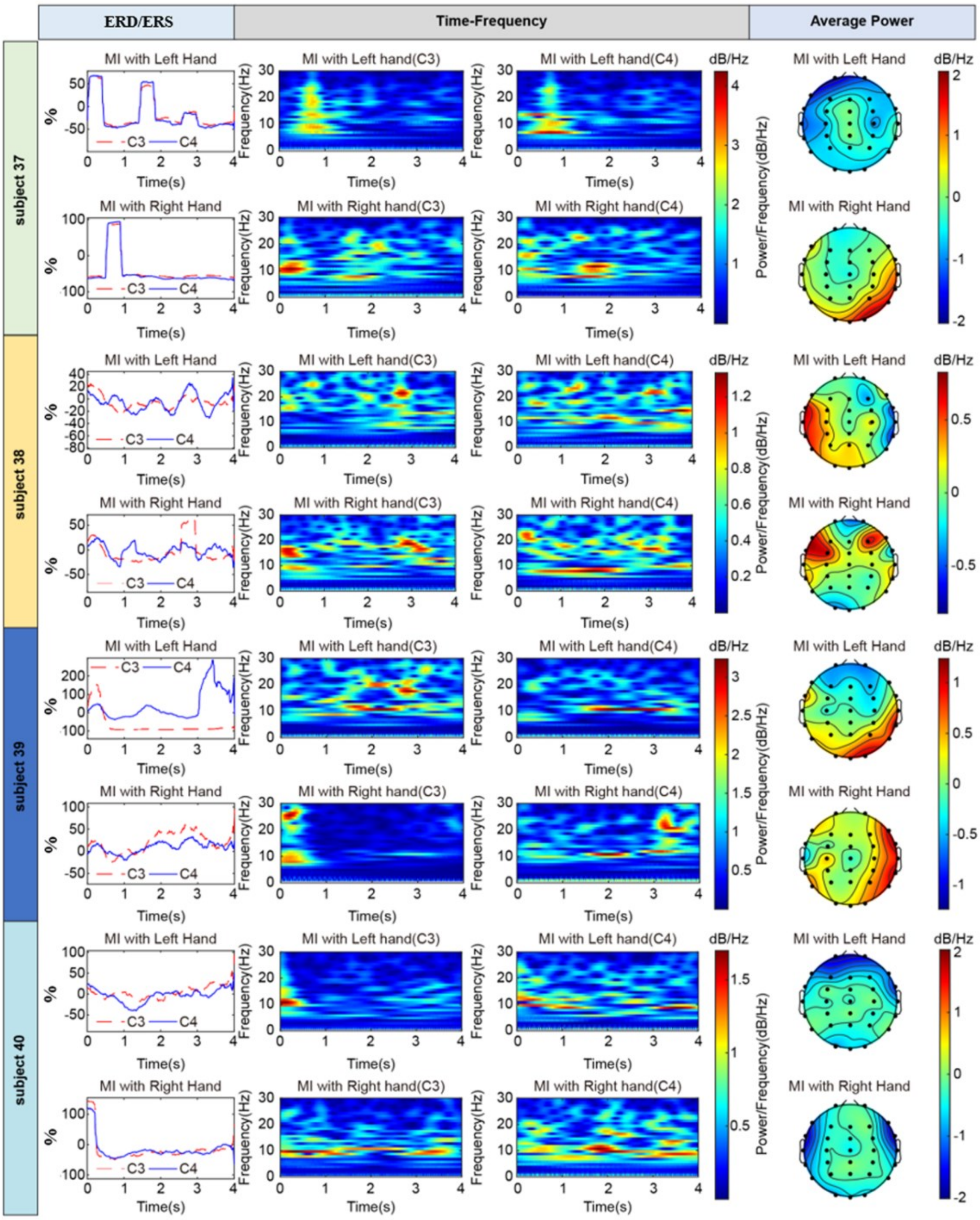

j. Result about ERD/ERS, time-frequency, and average power on the topmap of subjects 37-40

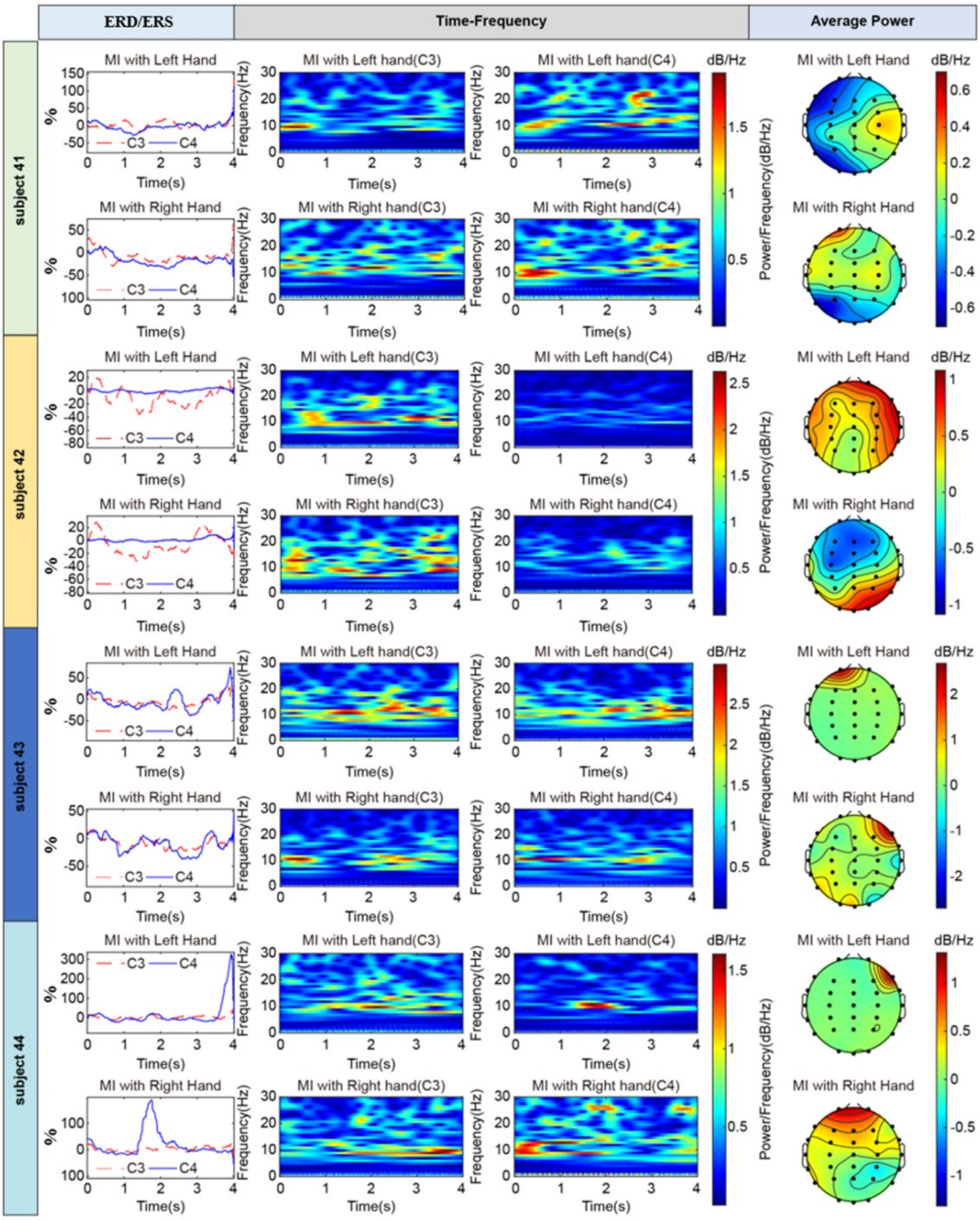

k. Result about ERD/ERS, time-frequency, and average power on the topmap of subjects 41-44

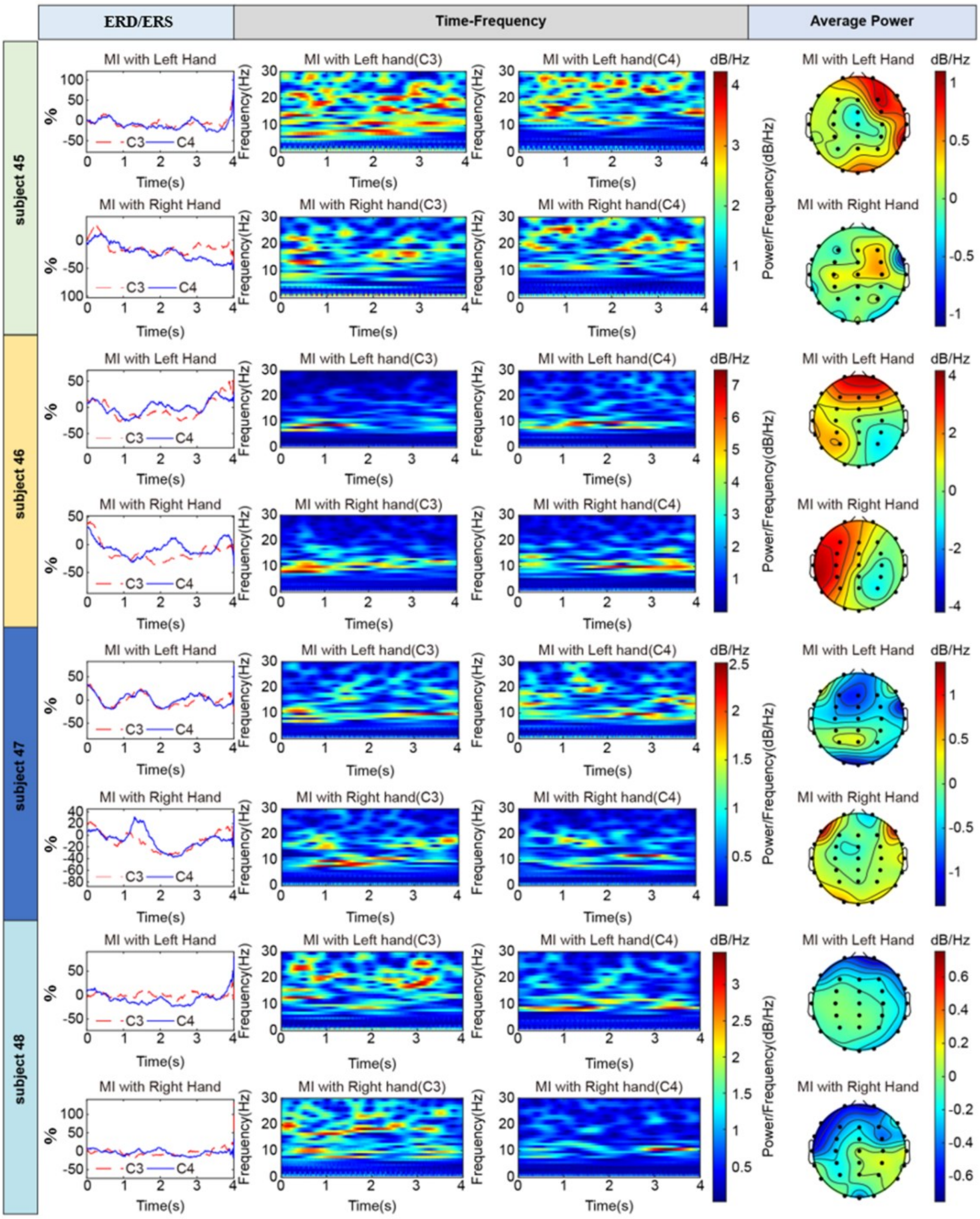

l. Result about ERD/ERS, time-frequency, and average power on the topmap of subjects 45-48

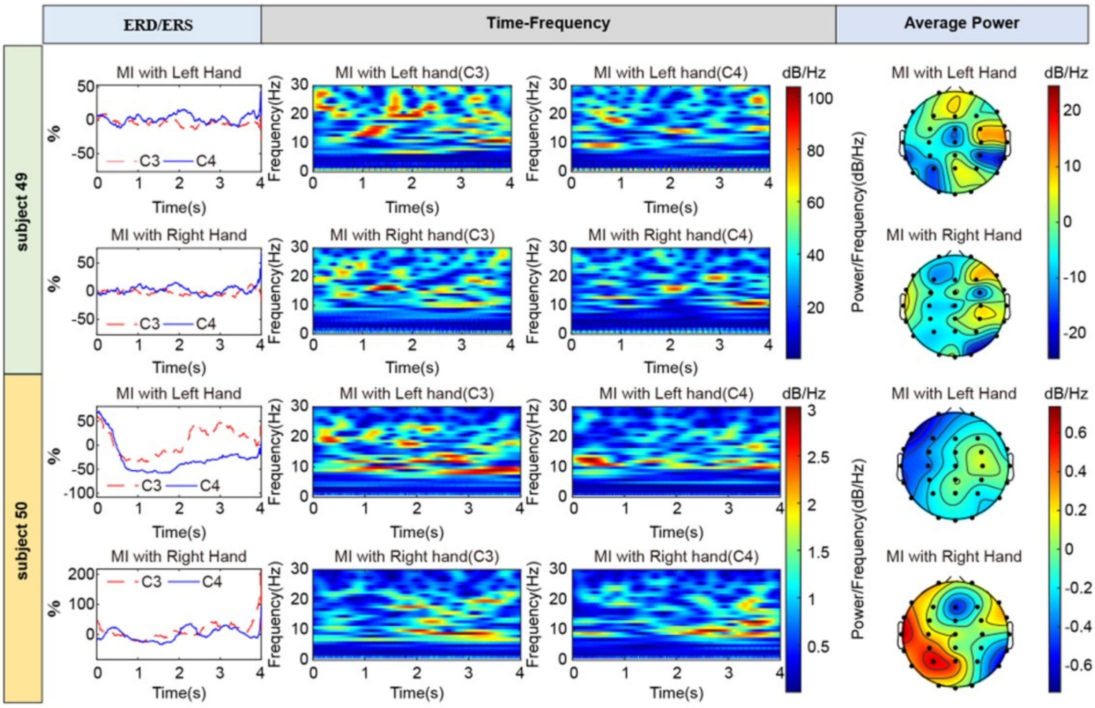

m. Result about ERD/ERS, time-frequency, and average power on the topmap of subjects 49-50
